# Validity and Reliability of the Stoma Self‐Efficacy Scale in Patients With Intestinal Stoma: A Descriptive, Cross‐Sectional and Validation Study

**DOI:** 10.1111/jocn.70168

**Published:** 2025-11-29

**Authors:** Lenka Machálková, Eva Reiterová, Karolína Křenková, Daniela Bartoníčková

**Affiliations:** ^1^ Department of Nursing, Faculty of Health Sciences Palacký University in Olomouc Olomouc Czech Republic; ^2^ Department of Anaesthesiology, Resuscitation and Intensive Medicine University Hospital Ostrava Ostrava‐Poruba Czech Republic

**Keywords:** factor analysis, intestinal stoma, reliability, stoma self‐efficacy scale, validity

## Abstract

**Aim(s):**

The aim was to validate the stoma self‐efficacy scale and assess the psychometric properties of the Czech version of the scale in patients with intestinal stoma. Another aim was to assess self‐efficacy in patients with intestinal stoma.

**Design:**

Descriptive, cross‐sectional and validation study.

**Methods:**

Two hundred and ninety patients with intestinal stoma participated in the study during 2023. The original SSES instrument was linguistically validated into Czech. Content validity of the scale, test–retest, intraclass coefficient, Cronbach's alpha, McDonald's *ω*, construct and convergent validity were assessed for psychometric properties. The study followed STROBE guidelines.

**Results:**

The stoma self‐efficacy scale was adopted into Czech, demonstrating excellent content validity. An intraclass correlation coefficient was calculated to establish test–retest reliability, showing excellent reliability of the Czech version. Cronbach's alpha and McDonald's *ω* showed high reliability. Factor analysis was applied for construct validity. Exploratory factor analysis was used to extract three factors on the Czech version of the scale: Stoma care self‐efficacy, social self‐efficacy and burden self‐efficacy. The factors accounted for 62.05% of the total variance and showed strong internal consistency. Confirmatory factor analysis was applied separately to the data of respondents with colostomy and respondents with ileostomy. The fit indices were satisfactory for respondents with colostomy after adjustment. The composite reliability coefficient showed acceptable values in each factor.

**Conclusion:**

The Czech version of the stoma self‐efficacy scale has excellent psychometric properties in patients with intestinal stoma. It is a reliable tool for use in patients with intestinal stoma to assess self‐efficacy. The scale can also be used by nurses who care for these patients and based on this, meet the individual needs related to patients' self‐efficacy.

## Abstract (highlights)

**Implications for the Profession(s) or Patient Care:**

This study was conducted to assess the level of self‐efficacy in intestinal stoma care. By validating the stoma self‐efficacy scale instrument to assess the level of self‐efficacy in intestinal stoma care for Czech patients, a Czech version of the scale was developed. The SSES scale can be used by patients and nursing staff (nurses) to determine the individual's needs in intestinal stoma care, in the possibility of maintaining social roles, contacts, activities of the individual and to assess stressful situations (including sexual), how the individual copes with them, possibly where and what specific help is needed. The scale's items provide structured support to help individuals lead a fulfilling life with intestinal stoma.

**Where and on Whom the Research Will Impact:**

- Patients—subjective assessment of self‐efficacy.
- Nurses—individual assessment and individual approach to patient problem solving.
- Opportunity to educate the patient, family, friends on problem areas of self‐efficacy.
- For the clinical setting—the possibility of using a valid SSES tool in stoma patients in the Czech setting.

**Patient Involvement in the Study:**

The study was conducted with patient participation. Patients were approached in stoma clinics of hospitals and stoma clubs. The research and their involvement in it were described, and their agreement to participation was voluntary. On involvement, they signed a written consent to participate in the research. The research was approved by the Ethics Committee of the Faculty of Health Sciences of Palacký University in Olomouc. Consent to carry out the research was obtained from the hospitals contacted in Moravia. Informed consent was obtained from patients in the stoma clubs involved who agreed to participate in the study.

**What Does This Article Contribute to the Wider Global Clinical Community?:**

- It highlights self‐efficacy as an important aspect in coping with living with an intestinal stoma, which may facilitate acceptance of an intestinal stoma and help living with it
- It encourages self‐efficacy assessment and fosters patient motivation by supporting its enhancement.
- Presents a reliable stoma self‐efficacy scale tool for Czech patients with intestinal stoma.

## 1 Introduction

The creation of an intestinal stoma is one of the common techniques of many surgical procedures in the treatment of colorectal cancer, a chronic inflammation of the intestine (Wang et al. 2018). In 2022, nearly 20 million new cases of cancer were diagnosed, with an estimated 35 million new cases by 2050, according to the International Agency for Research on Cancer (IARC). Globally, colorectal cancer is the third leading cause of new cases at 9.6% and the second leading cause of death at 9.3%. The highest incidence rates for colon cancer in both men and women are in Europe, Australia/New Zealand and North America. The incidence of rectal cancer is similar (Bray et al. 2024). In 2021, there were 6901 newly diagnosed cases of colorectal cancer in the Czech Republic (CR). This is 1.1% fewer than in the previous year. The male to female ratio in 2021 was 1.5:1. Colorectal cancer was the second leading cause of death for patients in the country in 2021, which was a 4.3% drop compared to the previous year. However, the incidence of colorectal cancer is steadily increasing. The prevalence is slightly higher in men than in women in the long‐term trend. The predominant age group is over 60 years old, with the highest prevalence in the 70–74 age group (Krejčí et al. 2022). Cancer presents a significant 21st‐century challenge across social, healthcare and economic sectors.

## 2 Background

The existence of intestinal stoma not only changes the bowel emptying habits of patients but is also a significant intervention in the lives of patients. It changes the patient's perception of the body (Karaçay et al. 2020). These changes can manifest in various aspects of life—for instance, patients often report limitations in clothing choices, diet and physical activities, as well as challenges in social interactions, intimate relationships or employment due to concerns about leakage, odour or stigma (Honkala and Berterö 2009; Thorpe et al. 2016; Sun et al. 2020). These changes are perceived in daily activities, lifestyle, work or personal life (Karaçay et al. 2020). In the Czech Republic, there are an estimated 14,000–15,000 patients with stomas (exact numbers are not recorded) (Czech ILCO 2025).

Self‐efficacy (SE) is of great importance to manage change, to cope with and adapt to living with a stoma (Su et al. 2016). Self‐efficacy allows for a personal assessment of one's ability to care for the stoma (Xu et al. 2018). Negatively, self‐efficacy may influence the self‐loathing, stigma experienced by patients (Jin et al. 2020). Adaptation to stoma increases as patients' level of self‐efficacy increases (Özden and Kılıç 2023). Supporting the patient in the process of adjusting to life with a stoma and accepting the stoma prevents stoma complications, reduces negative emotional reactions and is important to increase patient satisfaction and adaptation. Nurses who are actively involved in promoting the self‐efficacy of stoma patients in the postoperative period through professional interventions are of key importance (Bozkul et al. 2024). How patients manage stoma care is the subject of many studies in which various tools are used to assess the level of self‐efficacy (e.g., Bozkul et al. 2024; Alenezi et al. 2021). An adequate level of self‐efficacy is necessary, which can be determined by evaluating the stoma self‐efficacy scale (SSES), which has not been validated for the Czech clinical setting yet.

The validation of this instrument is essential, as cultural and healthcare system differences may influence how Czech patients perceive and express self‐efficacy. Individuals in the Czech Republic may access specialised stoma clinics, counselling services and stoma clubs, and ostomy supplies are reimbursed according to individual needs through the public health insurance system. According to findings by Wu et al. (2007) and Su et al. (2016), patients reported that ostomy supplies are expensive and that financial subsidies often do not fully cover their costs, forcing them to economise when purchasing higher‐quality products that enable social participation. The Czech nursing model, with its strong emphasis on nurse‐led education and long‐term follow‐up care, may shape patients' confidence in self‐care differently compared to other healthcare systems. Therefore, adapting and validating the SSES for the Czech context is important to ensure its conceptual, linguistic and cultural relevance for both clinical practice and research.

## 3 Aim

The aim of the study was to linguistically validate the original version of the 28‐item SSES, assess its validity, real‐world validity and psychometric properties for Czech patients with an intestinal stoma. Simultaneously, to determine self‐efficacy in patients with colostomy or ileostomy, including the effects of sociodemographic data and clinical characteristics of the stoma.

## 4 Methods

### 4.1 Design

A descriptive, cross‐sectional and validation study with linguistic validity of the SSES including content validity for Czech patients with intestinal stoma. The data obtained were psychometrically analysed.

### 4.2 Participants and Setting

Participants for the study were selected by purposive sampling, namely patients with intestinal stoma who attended the specialist stoma outpatient clinics of four hospitals or approached stoma clubs in Moravia, Czech Republic. Inclusion criteria for the study were: patients over 18 years of age, had been living with an intestinal stoma surgery (colostomy or ileostomy) for more than 1 year, spoke Czech, and expressed written consent to participate in the study. Patients under 18 years of age or with incomplete record sheets were excluded. The sample size was calculated using power analysis and according to Hendl (2004) (minimum number 255). Estimated sample size for the SSES questionnaire with 95% precision, Δ = 1.9; SD = 15.45 (Karaçay et al. 2020), calculated using the formula:

$$n={\left(\frac{{z_{1-}}_{\frac{\alpha }{2}}\mathrm{SD}}{\Delta }\right)}^{2}={\left(\frac{1.96\times 15.45}{1.9}\right)}^{2}=254.3$$

The required sample size was determined to be 255 respondents. Moreover, according to the rule of 10 respondents to item, the sample of 290 respondents was considered sufficient for the analysis of 28‐item scale (Hendl 2017). For the test–retest, the sample size was set at 27 respondents (Hendl 2004).

### 4.3 Data Collection

**FIGURE 1:**
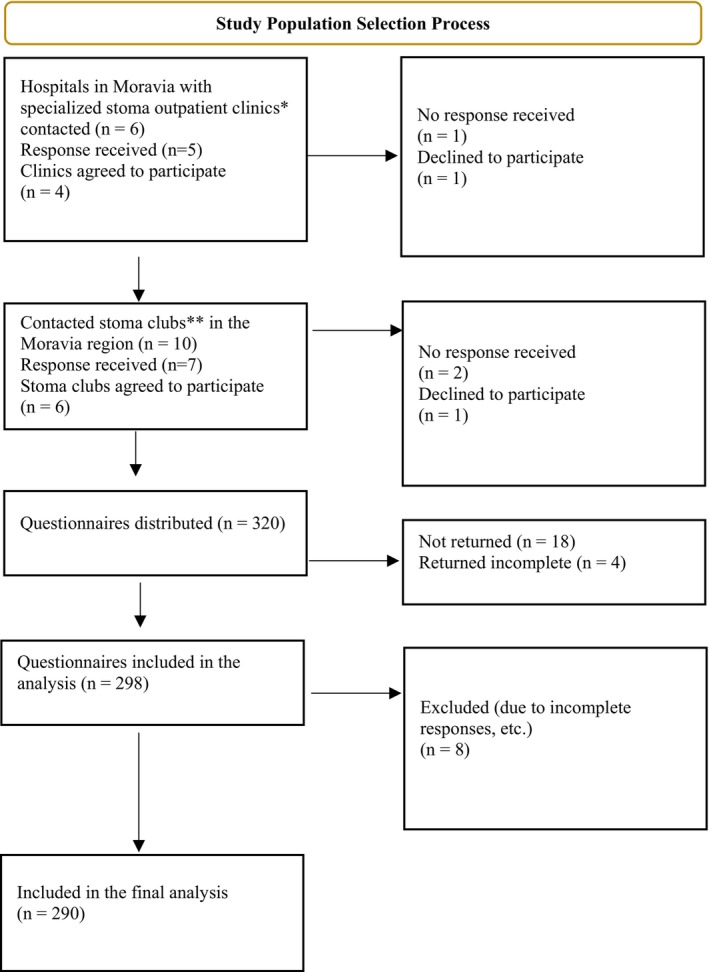
Flow diagram of the study population selection process. *Stoma outpatient clinics are specialised clinics for patients with stomas, providing information on comprehensive stoma care, diagnostics, treatment and prevention of complications with stoma insertion. It offers counselling for the patient and family members in practical stoma care, provision of aids, nutritional and social counselling. **Stoma clubs are voluntary associations of individuals with a stoma. Group meetings offer individuals the opportunity to regain their often‐lost self‐confidence and new energy for life in society. The meetings include discussions with experts, representatives of companies dealing with stomy issues (e.g., ILCO, Convatec), presentations of new devices and information on the possibilities of using them. The interaction of individuals with stoma is of great importance in the personal experiences. [Colour figure can be viewed at wileyonlinelibrary.com]

Data was collected in 2023, when patients were approached by stoma nurses in stoma clinics. In the stoma clubs, personal contact with the patient was made directly by the researcher (Figure 1).

#### 4.3.1 Data Recording Sheet

After consent was given, the patient was given a record sheet containing the SSES scale and additional data. To obtain supplementary data, an 11‐item questionnaire with patient sociodemographic data (age, sex, marital status, education, economic status, social status) and clinical characteristics of the stoma (type of stoma, duration of stoma insertion, age at stoma insertion, cause of insertion, perception of stoma) was developed by the authors for this study.

The SSES scale was developed by Bekkers et al. (1996) to test the self‐efficacy of stoma patients. The scale contains 28 items. The scale items are rated on a 5‐point Likert scale (1 = not at all confident, 5 = totally confident). Possible scores on the scale range from 28 to 140. The higher the scale score, the better the level of self‐efficacy. The original scale contains two subscales. The stoma care self‐efficacy subscale (stoma care SE) measures the ability to care for one's own stoma and contains 13 items (e.g., item no. 3: Take care of the stoma in the right way at home.). The nine items in the second subscale, social self‐efficacy (social SE), measure social functioning with a stoma (e.g., item no. 18: Tell other people about the stoma). In the original scale, the Cronbach's alpha of the stoma care SE was 0.94 and the social SE was 0.95. The correlation between stoma care SE and social SE was high (*r* = 0.73). The scale also contains six items that the authors believe were not included in the subscales because of response bias or low item‐level intercorrelations (Bekkers et al. 1996).

#### 4.3.2 Language Validity of the SSES

The linguistic adaptation of the scale to the Czech environment was carried out according to the methodology of Repo and Rosqvist (2016), Sousa and Rojjanasrirat (2011) and the WHO recommendations (2020). At the beginning of the process, translators and stakeholders were approached and their consent was obtained. Three translations were done by independent translators into the Czech language (professional translator, native speaker, expert in the field). Subsequently, a comparison (professional translator, researcher, practitioner) and a synthesis of the three Czech versions was made. This was followed by back‐translation from Czech to English by three independent translators (professional translator, native speaker, practitioner) and three English versions were obtained. The translated versions were compared and evaluated with the original tool by the researcher, the domain expert, the translator. Then, the working Czech version of the SSES scale was aligned by a team of experts (researcher, nursing academic, translator). Cognitive debriefing was performed to determine the level of comprehensibility and cognitive equivalence of the translation of the scale in 10 Czech adult patients with stoma. Based on their comments, minor modifications were made (e.g., item 6 used ‘stoma collection materials’ and was replaced with a Czech equivalent of ‘aid/device’ in the translation). In addition, the translation of the scale in Czech was proofread and edited (for stylistic and linguistic correctness). The numbering of items (1–28) and the final correction of the scale was carried out by a university expert in nursing and a university expert in the Czech language.

#### 4.3.3 Content Validity of the Scale

Content validity was assessed by three university‐educated nursing practitioners (two stoma nurses, a surgeon) on a 4‐point scale (1 = not relevant, 2 = somewhat relevant, 3 = completely relevant, 4 = highly relevant). The experts independently rated each scale item according to its relevance. They could also give suggestions for change if needed. The final Czech version of the scale was created and used for pilot testing of the clarity of the scale with eight patients with intestinal stoma and eight professionals (stoma nurses) who rated on a 3‐point scale (1 item unclear, 2 items clear, 3 items very clear). There was no change necessary after testing and thus the items were considered clear (Polit et al. 2007, 2012). A content validity index (CVI) was calculated based on the established ratings.

#### 4.3.4 Pre‐Test Scale Reliability

The reliability of the Czech version of the scale was assessed through individual analysis of the 28 items, internal consistency and test–retest.

The test–retest was conducted on a pilot sample of 27 patients with intestinal stoma who attended stoma clinics of hospitals (20 patients) and stoma clubs (7 patients). Patients were tested twice within 14 days, according to the recommended interval of 1–2 weeks (Polit 2014). Test–retest reliability reflects the variation of measurements made with the same measuring instrument on the same group of people under approximately the same conditions at a certain time interval. To determine test–retest reliability, the intraclass correlation coefficient (ICC) was calculated, which is widely used as a reliability index in test–retest reliability analyses. It reflects both the degree of correlation and agreement between the measurements. A value greater than 0.9 indicates excellent reliability of the instrument, values between 0.75 and 0.9 indicate good reliability, values between 0.5 and 0.75 indicate moderate reliability, and values less than 0.5 indicate poor reliability (Koo and Li 2016).

The test–retest reliability was assessed using ICC, calculated according to McGraw and Wong's definition (Koo and Li 2016). A two‐way mixed‐effects model (absolute agreement, average measures) was applied to evaluate the degree of consistency between repeated administrations of the SSES. The ICC value was reported overall with its 95% confidence interval to provide an estimate of measurement precision.

All 28 original items were included and analysed for the Czech version.

### 4.4 Data Analysis

Data were analysed and subsequently processed using Microsoft Excel, IBM SPSS Statistics version 30 and IBM SPSS Amos version 30.

#### 4.4.1 Reliability

Test–retest reliability was assessed on the pilot sample using ICC, Cronbach's alpha (DeVellis 2022) and paired *t*‐test.

Cronbach's alpha and McDonald's *ω* were calculated on the entire sample of patients for all questionnaire items. Next, item analysis was performed with the calculation of Cronbach's alpha after removing an item from the scale and correlating each item with the total score. The reliability of the individual subscales was also calculated (George and Mallery 2021).

#### 4.4.2 Construct and Convergent Validity

The construct validity of the entire questionnaire was verified by exploratory factor analysis (EFA) followed by confirmatory factor analysis (CFA) for respondents with colostomy and respondents with ileostomy (Field 2024; Kline 2023).

The convergent validity of the SSES was calculated using the composite reliability (CR) coefficient and the average variance extracted (AVE). According to Shrestha (2021), CR and AVE values range from 0 to 1, with higher values indicating greater reliability.

#### 4.4.3 Scale Analysis

Basic descriptive statistics were used in the statistical analysis with calculations of frequencies (*n*) and percentages (%), means and standard deviations (SD). The total SE score of patients was evaluated according to their level of self‐efficacy: a low level was defined as a score of ≤ 65 points, a moderate level as a score between 66 and 102 points, and a high level as a score ≥ 103 points (Wang et al. 2022). Associations with sociodemographic variables were examined, including gender, education, social, economic and family status, as well as the cause and perception of the stoma. Between‐group differences were tested using Student's *t*‐test. Associations of numerical variables with each subscale and the total score—including the subgroup of respondents with a stoma—were assessed using Spearman's correlation coefficient.

### 4.5 Ethical Aspect

The approval of the Ethics Committee was obtained for the implementation of the research (Approval No. UPOL‐253378/FZV‐2022). Consent was obtained for the implementation of the research in the participating hospitals in Moravia; informed consent was obtained from patients in the participating stoma clubs who agreed to be involved in the study. Participation in the study was voluntary, and patients did not incur any obligations or claims.

## 5 Results

### 5.1 Sociodemographic Characteristics of Respondents With Intestinal Stoma

In the sample of 290 participants, 45.52% (*n* = 132) were male and 54.48% (*n* = 158) were female, aged between 21 and 89 years, with a mean age of 59.97 years (SD = 15.67). The majority were married (66.55% (*n* = 193)), 34.45% (*n* = 97) were single, 55.52% (*n* = 161) had a secondary education, 36.21% (*n* = 105) had a tertiary education. 72.42% (*n* = 210) of respondents were old age pensioners, 21.72% (*n* = 63) received a salary and 18.97% (*n* = 55) lived alone. They had been living with an intestinal stoma for a mean of 7.66 years (SD = 8.01), ranging from 1 to 40 years living with a stoma. The mean age of respondents at the time of stoma insertion was 52.30 (SD = 15.87) years. 60.69% (*n* = 176) had colostomy and 39.31% (*n* = 114) had ileostomy, in 55.52% (*n* = 161) for oncological reasons. The mean age of respondents with a colostomy was 63.92 (SD = 13.81) years, and they had been living with a colostomy for a mean of 7.71 (SD = 7.53) years, with a mean age of insertion of 56.21 (SD = 12.69) years. The mean age of the respondents with ileostomy was about 10 years lower at 53.86 (SD = 16.45) years than that of the respondents with a colostomy. Also, the mean age at ileostomy insertion was about 10 years lower in respondents with ileostomy, namely 46.27 (SD = 18.28) compared to respondents with colostomy. The ileostomy insertion age of respondents was 7.59 (SD = 8.78) years on average. Considering the above findings, the data were further analysed for respondents with colostomy and respondents with ileostomy. Detailed socio‐demographic data for respondents with colostomy and respondents with ileostomy are presented in Table 1.

**TABLE 1:** Descriptive characteristics of colostomy (*n* = 176) and ileostomy (*n* = 114) participants.

Variable	Category	Respondents with colostomy	Respondents with ileostomy	Respondents with stomy
		*n* (%)	*n* (%)	*n* (%)
Sex	Male	89 (50.57)	43 (37.72)	132 (45.52)
	Female	87 (49.43)	71 (62.28)	158 (54.48)
Education	Primary school	15 (8.52)	9 (7.89)	24 (8.27)
	Secondary school	99 (56.25)	62 (54.39)	161 (55.52)
	Tertiary	62 (35.23)	43 (37.72)	105 (36.21)
Social status	Lives alone	40 (22.73)	15 (13.16)	55 (18.97)
	Lives with someone	136 (77.27)	99 (86.84)	235 (81.03)
Economic status	Receives a salary	30 (17.05)	33 (28.95)	63 (21.72)
	Receives a pension	135 (76.70)	75 (65.79)	210 (72.42)
	Social support	11 (6.50)	6 (5.27)	17 (5.86)
Marital status	Married	121 (68.75)	72 (63.16)	193 (66.55)
	Single	55 (31.25)	42 (36.84)	97 (34.45)
Cause	Bowel or rectal cancer	131 (74.44)	30 (26.32)	161 (55.52)
	Inflammation of the intestine	45 (25.57)	84 (73.68)	129 (44.48)
Stoma perception	Positive	120 (68.18)	84 (73.68)	204 (70.34)
	Negative	56 (31.82)	30 (26.32)	86 (29.66)

Variable	Mean (SD)	Mean (SD)	Mean (SD)
Age of respondents	63.92 (13.81)	53.86 (16.45)	59.97 (15.67)
Age of respondents at stoma insertion	56.21 (12.69)	46.27 (18.28)	52.30 (15.87)
Duration of stoma insertion	7.71 (7.53)	7.59 (8.78)	7.66 (8.01)

*Note: n*, number of participants included in the analysed sample.

### 5.2 Psychometrics of the Czech Version of the SSES

#### 5.2.1 Content Validity of the Czech Version of the SSES

The CVI of the Czech version of the scale was calculated as 0.94, which is excellent content validity.

#### 5.2.2 Test–Retest Reliability of the Czech Version of the SSES

The SSES scale was evaluated for reliability using the test–retest method in 27 patients with intestinal stoma.

All methods were applied to all 28 items of the scale. An ICC = 0.95 was found for 95% reliability (confidence interval 0.89–0.98) for all items. The correlation between the sums of the first and second measurements was high at 0.95 and significant (*p* < 0.001). Cronbach's alpha for the first measurement was 0.95 and 0.97 for the second measurement. The result of the paired *t*‐test (*t* = 0.12) showed that there is no significant difference (*p* = 0.903) between the mean scores (mean of the first measurement 108.04 (SD = 19.1); mean of the second measurement was 107.37 (SD = 20.73)).

#### 5.2.3 Reliability of the Czech Version of the SSES (*N* = 290)

The reliability of the scale was calculated using Cronbach's alpha (= 0.94) and McDonald's *ω* (= 0.95). The reliability of each subscale is presented in Table 2. The results showed high reliability. In addition, Cronbach's alpha SSES for respondents with colostomy (0.94) and respondents with ileostomy (0.93) and McDonald's *ω* SSES for respondents with colostomy (0.94) and respondents with ileostomy (0.93) were calculated. Within each factor, Cronbach's alpha ranged from 0.80 for stoma care SE, 0.88 for Burden SE and 0.95 for Social SE for respondents with colostomy. For respondents with ileostomy, it ranged from 0.85 for Social SE, 0.86 for Burden SE and 0.94 for stoma care SE.

**TABLE 2:** Item analysis, exploratory factor analysis with Oblimin rotation and convergent validity of the stoma self‐efficacy scale (*n* = 290).

Item	Mean	SD	Correlation	F1	F2	F3
1	3.84	1.08	0.73	**0.56**	0.16	0.22
2	3.55	1.12	0.62	**0.43**	0.08	0.30
3	4.24	1	0.68	**0.7**	0.03	0.15
4	3.79	2.6	0.32	**0.17**	0.17	0.04
5	3.63	1.04	0.72	**0.57**	0.15	0.17
6	4.27	0.95	0.69	**0.78**	0.07	0.03
7	3.68	1.12	0.55	**0.6**	0.24	−0.17
8	4.39	0.82	0.50	**0.87**	−0.13	−0.08
9	4.18	0.91	0.48	**0.84**	−0.13	−0.08
10	3.93	1.08	0.73	**0.68**	0.18	0.06
11	3.77	1.12	0.70	**0.65**	0.16	0.07
28	4.06	1.06	0.70	**0.59**	0.06	0.27
12	3.86	1.06	0.71	0.29	**0.48**	0.08
14	3.97	0.99	0.63	0.16	**0.46**	0.13
15	3.78	1.24	0.76	0.2	**0.62**	0.07
16	3.65	1.27	0.72	0.05	**0.63**	0.17
17	3.86	1.26	0.62	0.05	**0.86**	−0.18
18	3.34	1.41	0.56	−0.09	**0.89**	−0.13
19	4.13	1.06	0.79	0.17	**0.67**	0.09
20	3.75	1.27	0.79	−0.00	**0.72**	0.24
21	3.6	1.36	0.74	0.01	**0.78**	0.09
22	3.01	1.43	0.72	−0.07	**0.80**	0.14
23	3.19	1.41	0.77	0.01	**0.59**	0.35
24	2.68	1.54	0.60	−0.03	0.02	**0.88**
25	2.63	1.5	0.58	−0.02	−0.03	**0.91**
26	2.98	1.37	0.67	0.09	0.17	**0.62**
27	3.02	1.33	0.67	0.18	0.11	**0.61**
13	4.39	3.15	0.23	0.21	0.00	0.1
Eigenvalue with item 13				3.25	2.14	1.44
Eigenvalue without item 13				13.19	2.13	1.43
Explained cumulative variance with item 13				47.33	54.95	60.08
Explained cumulative variance excluding item 13				48.85	56.74	62.05
Cronbach's *α* without item 13				0.88	0.88	0.87
Cronbach's *α* with item 13				0.82	—	—
McDonald's *ω* without item 13				0.88	0.88	0.85
McDonald's *ω* with item 13				0.82	—	—
AVE without item 13				0.24	0.23	0.15
AVE with item 13				0.23	—	—
CR without item 13				0.73	0.72	0.62
CR with item 13				0.71	—	—

*Note:* Bold values indicate the factor loading of items and represent assignment to the factors.

Abbreviations: AVE, average variance extracted; CR, composite reliability; F1, stoma care SE; F2, social SE; F3, burden SE; SD, standard deviation; SE, self‐efficacy.

#### 5.2.4 Item Analysis of the Czech Version of the SSES

An item analysis of the SSES scale was conducted (Table 2). Item 13 (Perform light housework) was removed due to a low correlation value (0.23). The control calculation showed no statistically significant difference (*p* = 0.346) in the total score of the Czech version of the SSES without item 13 for respondents with colostomy, nor for respondents with ileostomy. The obtained values (eigenvalues, cumulative variance) showed better results when item 13 was excluded (Table 2).

#### 5.2.5 Exploratory Factor Analysis to Determine the Factor Structure of the SSES

Factor analysis was applied to establish the construct validity of the scale. EFA was conducted for a set of 290 respondents with intestinal stoma, CFA for 176 respondents with colostomy and 114 respondents with ileostomy.

Prior to the EFA, data were tested with the Kaiser–Meyer–Olkin measure (0.931) and Bartlett's test of sphericity (Chi‐square = 6330, *p* < 0.001) to establish suitability. These values indicate that EFA can be applied (Jamil et al. 2018). Principal component analysis with Oblimin rotation was performed as part of the EFA. Three factors were extracted based on the eigenvalues (> 1) (Table 2). Item 13 (perform light housework) was not included in any of the three identified factors due to low factor loadings (Table 2). After removing item 13, the cumulative percentage of variance increased from 60.08 to 62.05 (Table 2). The factor loadings for each component (item and factor correlations) are presented in Table 2. Factor 1, labelled stoma care self‐efficacy (stoma care SE), items 1–11 and 28, focus on and measure individual ability to care for their stoma. Factor 2 labelled social self‐efficacy (social SE), items 12, 14–23, measure individuals' abilities in their social functioning. Factor 3, labelled self‐efficacy in burden situations (burden SE), includes items 24–27 that measure an individual's abilities in personal burden situations and activities. Cronbach's alpha and McDonald's *ω* for each factor are shown in Table 2.

#### 5.2.6 Confirmatory Factor Analysis

**FIGURE 2:**
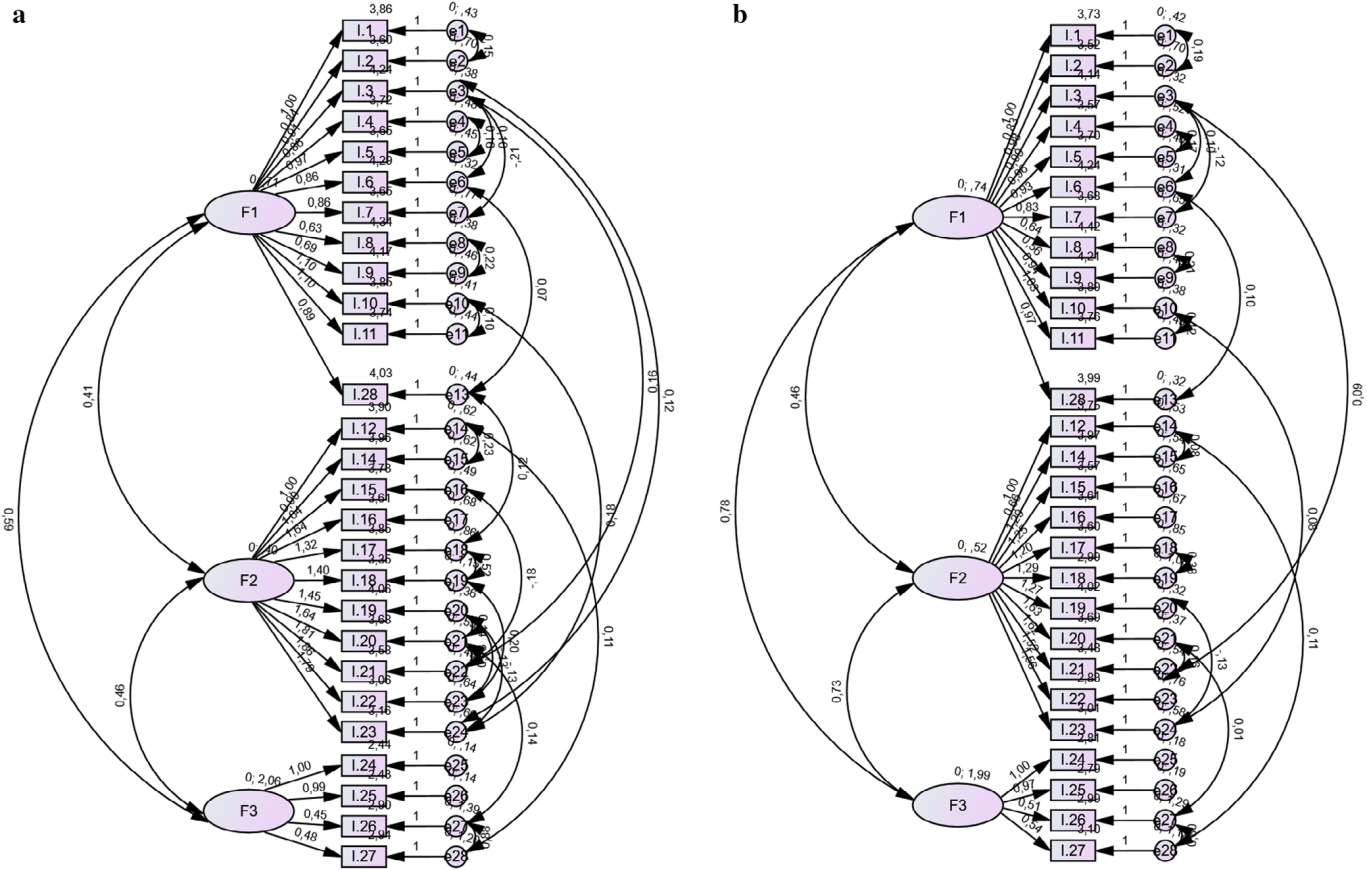
Confirmatory factor analysis of SESS. (a) Three‐factor solution for respondents with colostomy. (b) Three‐factor solution for respondents with ileostomy. [Colour figure can be viewed at wileyonlinelibrary.com]

The CFA separately examined the fit index of the three factors solution of the SSES scale with the data for respondents with colostomy and respondents with ileostomy. It was found that all fit indices for respondents with colostomy were satisfactory after adjustment using modified indices. An RMSEA value < 0.05 is a close fit of the model to the data, up to 0.08 is a reasonable fit, and > 1 is not recommended. In the model of this study, RMSEA = 0.07 was a good fit only for respondents with colostomies. The CMIN/df and NFI values were appropriate for both models. For the CFI, IFI indices, a goodness of fit of 0.8–0.9 is acceptable. The NFI and TLI values for respondents with ileostomies did not reach the desired value (Table 3). In Table 3, the compliant values for both models are shown in bold. Factorial solutions for respondents with colostomy and respondents with ileostomy are depicted in Figure 2a,b. During model modification, adjustment lines were added to the graphs (Figure 2a,b) based on modification indices. These lines denote pairwise correlations between items in the best‐fitting model. Among respondents with colostomies or ileostomies, a noteworthy correlation was observed between item 10 (Caring for the stoma outside the home) and item 23 (Being able to go on vacation as before stoma formation), which was stronger in the colostomy subgroup (*ρ* = 0.18) than in the ileostomy subgroup (*ρ* = 0.08). Likewise, the correlation between item 3 (Caring for the stoma correctly at home) and item 21 (Spending the night away from home with friends who know about the stoma) was higher among respondents with colostomies (*ρ* = 0.16) than among those with ileostomies (*ρ* = 0.09). These effects are small in magnitude.

**TABLE 3:** Fit indices.

Fit index	Desired value	Respondents with colostomy	Respondents with ileostomy
CMIN/df	< 3	**1.95**	**2.16**
CFI	> 0.9	**0.93**	0.87
IFI	> 0.9	**0.93**	0.87
TLI	> 0.9	**0.91**	0.73
NFI	> 0.8	**0.86**	0.78
RMSEA	< 0.08	**0.07**	0.1

*Note:* Bold values indicate the factor loading of items and represent assignment to the factors.

Abbreviations: CFI, comparative fit index; CMIN/df, Chi‐square divided by degrees of freedom; IFI, incremental fit index; NFI, normed fit index; RMSEA, root mean square error of approximation; TLI, Tucker–Lewis index.

For respondents with colostomies, an additional correlation (*ρ* = 0.14) was found between ‘The ability to go to a restaurant or cinema as before stoma formation’ (item 20) and ‘Performing more demanding household or outdoor work’ (item 26). Another correlation (*ρ* = 0.12) was observed between ‘Caring for the stoma properly at home’ (item 3) and ‘Going on vacation as before stoma formation’ (item 23). These findings suggest that such situations reflect respondents' need to ensure an adequate supply of stoma appliances and access to appropriate hygiene facilities for stoma care. These factors appear particularly salient when being or moving outside the home, whereas at home respondents generally feel safe and well‐equipped for stoma care.

#### 5.2.7 Convergent Validity

Convergent validity of the Czech version of the SSES scale was assessed using the CR and AVE coefficients. For the three factors structured in this study, the CR values were found to be 0.73 for stoma care SE, CR = 0.72 for Social SE and CR = 0.62 for Burden SE. AVE values were found to be less than 0.5 (Table 2). Fornell and Larcker (1981) stated that if AVE is less than 0.5 but CR is greater than 0.6, convergent validity is acceptable.

### 5.3 Results of the Self‐Efficacy Assessment in Respondents With Intestinal Ostomies

#### 5.3.1 Perception of Intestinal Stoma and Level of Self‐Efficacy

70.34% (*n* = 204) had a positive perception of stoma and 29.66% (*n* = 86) had a negative perception of stoma (Table 1). For respondents with stomas, the mean SE score was 98.71 (SD = 22.5), ranging from 35 to 135. For respondents with colostomy, it was 97.84 (SD = 22.18), ranging from 35 to 135 points, and for respondents with ileostomy, it was higher at 100.05 (SD = 23.02), ranging from 37 to 135 points.

High level of self‐efficacy was found in both respondents with colostomy (11.93%) and respondents with ileostomy (11.40%). Medium level of self‐efficacy was prevalent in respondents with both ileostomy and colostomy. Low level of self‐efficacy was reported by 32.38% of respondents with colostomy (Table 4).

**TABLE 4:** Level of self‐efficacy in respondents with stomas.

Respondents	Self‐efficacy			
	Low	Medium	High	Total
	*n* (%)	*n* (%)	*n* (%)	*n* (%)
With ileostomy	29 (25.45)	72 (63.15)	13 (11.40)	114 (100)
With colostomy	57 (32.38)	98 (55.68)	21 (11.93)	176 (100)
With stomy	86 (29.66)	170 (58.62)	34 (11.72)	290 (100)

*Note: n*, size of the processed part of the sample group; Low, total score < 65, medium, 66–102, high, total score > 103.

#### 5.3.2 Associations With Socio‐Demographic Data

Analysis of the data in the cohort of respondents with stoma revealed associations for the variables: gender, economic status and perception of stoma. Social status, marital status, cause and education level were not significant variables in this cohort (Table 5).

**TABLE 5:** Student‘s *t*‐test and analysis of variance for the set of respondents with ileostomy and respondents with colostomy.

Student's *t*‐test							
Variable	Category	Respondents with ileostomy			Respondents with colostomy		
		Average	*t*	*p*	Average	*t*	*p*
Gender	Male	102.88	−0.866	0.388	101.53	2.256	0.025
	Female	98.93			94.07		
Social status	Lives alone	93.53	1.216	0.227	93.85	−1.297	0.196
	Living with someone	101.46			99.02		
Marital status	Married	102.14	−1.018	0.311	98.92	0.959	0.339
	Single	97.48			95.43		
Cause	Bowel cancer, rectum	93.50	1.893	0.061	97.08	−1.103	0.272
	Inflammations of the intestine	102.89			104.25		
Stoma perception	Positive	107.79	−6.537	*p* < 0.001	103.90	−5.774	*p* < 0.001
	Negative	79.77			84.86		

Analysis of variance			*F*	*p*		*F*	*p*
Education	Elementary	87.67	1.106	0.35	95.73	2.237	0.086
	Secondary	102.16			101.06		
	Tertiary education	100.91			102.30		
Economic status	Salary	108.82	3.302	0.023	107.20	3.399	0.019
	Old‐age pension	97.79			96.87		
	Other	80.60			83.13		

*Note: t*, Student‘s *t*‐test.

In the present study, respondent age within each subscale was negatively correlated (*r* = −0.18) with total SE score (*p* = 0.002), indicating that as respondent age increases, total SE score decreases, especially in stressful and sexual activities (burden SE; Table 6).

**TABLE 6:** Correlation of age of respondents with stoma with total score and in each subscale.

Variable	Stoma care SE	Social SE	Burden SE	Total score
	*r* (*p*)	*r* (*p*)	*r* (*p*)	*r* (*p*)
Age of respondents	−0.13 (*p* = 0.027)	−0.10 (*p* = 0.105)	−0.36 (*p* < 0.001)	−0.18 (*p* = 0.002)
Age of respondents at stoma insertion	−0.19 (*p* < 0.001)	−0.21 (*p* < 0.001)	−0.42 (*p* < 0.001)	−0.28 (*p* < 0.001)
Duration of stoma insertion (years)	0.22 (*p* < 0.001)	0.24 (*p* < 0.001)	0.12 (*p* = 0.042)	0.23 (*p* < 0.001)

*Note: r*, Spearman's rank coefficient.

Abbreviation: SE, self‐efficacy.

Furthermore, it was found that the SE score increases with increasing duration of the stoma insertion (*r* = 0.25; *p* < 0.001), especially in respondents with ileostomy (Table 7). When the stoma was inserted at a younger age of the respondent, it can be expected to have a higher SE (*r* = −0.24; *p* < 0.001) (Table 7).

**TABLE 7:** Correlation of respondent age, age at stoma insertion and duration since stoma insertion in the cohort of respondents with stomas.

Variable	Respondents with ileostomy	Respondents with colostomy	Respondent with stomy
	*r* (*p*)	*r* (*p*)	*r* (*p*)
Age of respondents	−0.23 (*p* = 0.012)	−0.10 (*p* = 0.184)	−0.17 (*p* = 0.004)
Age of respondents at stoma insertion	−0.4 (*p* < 0.001)	−0.19 (*p* = 0.011)	−0.24 (*p* < 0.001)
Duration of stoma insertion (years)	0.38 (*p* < 0.001)	0.15 (*p* = 0.043)	0.25 (*p* < 0.001)

*Note: r*, Spearman's coefficient.

## 6 Discussion

The study aimed to assess SE in patients with intestinal stomas using the SSES. The SSES was linguistically validated for Czech patients with colostomies and ileostomies. Internal consistency was excellent with Cronbach's *α* and McDonald's *ω* > 0.9 for the total SSES and > 0.8 across individual factors. Test–retest reliability demonstrated high stability of the Czech version of the SSES with ICC = 0.95.

Moderate self‐efficacy was prevalent across colostomy and ileostomy patient subgroups. In the subgroup with colostomies, 32.38% of patients showed low SE. Self‐efficacy declined with increasing age, particularly in stressful situations and sexual activities, while longer stoma duration was associated with higher SE. It can be assumed that patients with stoma insertion at a younger age tend to demonstrate higher SE.

### 6.1 Comparison of the Psychometric Properties of the SSES

Self‐efficacy is a prerequisite for success in an individual's stoma care. Evaluating self‐efficacy and determining the level of self‐efficacy is important to enhance the quality of life of an individual with a stoma. To do this, valid and reliable tools are necessary. Bekkers et al. developed the SSES assessment tool in 1996, which has been validated and subsequently used in various countries around the world to assess the level of self‐efficacy in patients with stomas. In Turkey (Karaçay et al. 2020), the SSES was evaluated as a valid and reliable instrument for Turkish stoma patients based on the validation of 22 items with two subscales. In the Turkish version, the reliability level of the CVI was excellent. In construct validity the two factors explained 60.29% of the total variance. The Cronbach's alpha for internal consistency and the ICC value for the scale were adequate (Karaçay et al. 2020). Also, in the Chinese version of the SSES scale (Wu et al. 2007), the Cronbach's alpha for the 22 items was highly acceptable. The Iranian version of the SSES scale (Nasiriziba et al. 2020) was assessed in terms of the reliability of the 28‐item scale using Cronbach's alpha (0.9). The Cronbach's alpha for the Czech version of the 28‐item scale was also satisfactory. In this study, the Czech version of the SSES scale for stoma patients was assessed as valid and reliable. The CVI and ICC value of the Czech version was conformable like the Turkish version (CVI = 0.96; ICC = 0.97) (Karaçay et al. 2020). The Indonesian version validated only the 13‐item stoma care self‐efficacy (stoma care SE) scale. Items were rated on a 5‐point Likert scale (1—not confident, 5—extremely confident) and total scores ranged from 13 to 65. The content validity value of the CVI was highly acceptable. The reliability of the 13‐item scale assessed using Cronbach's alpha was also adequate (Zainuddin et al. 2020), however lower than in the Turkish (0.97) (Karaçay et al. 2020) and Chinese (0.97) versions (Wu et al. 2007) of stoma care SE.

In the Czech validation, a three‐factor structure emerged instead of the original two‐factor model, suggesting that patients distinguish practical stoma care, social confidence and coping in stressful or intimate situations as separate domains of self‐efficacy. This likely reflects Czech nursing practice, which emphasises patient autonomy and individual self‐care. Such differentiation may also stem from cultural differences in discussing sensitive topics, such as sexuality or body image. During validation, Item 13 (‘performing light housework’) was removed due to a low correlation coefficient and weak factor loading. From a clinical perspective, this activity may not represent a core aspect of self‐efficacy for Czech stoma patients, as many receive family or home‐care assistance and associate confidence mainly with self‐care and stoma management rather than household work. The model showed a good overall fit for the colostomy subgroup but a weaker fit for the ileostomy subgroup. This difference may reflect the greater physiological complexity and dietary demands associated with ileostomy care, which could lead to more heterogeneous perceptions of self‐efficacy and slightly reduced model stability.

### 6.2 Self‐Efficacy Findings in Stoma Patients in Comparison to Other Studies

This study included all 28 items of the SSES scale. The items measure self‐efficacy in stoma care, social domains, as well as sexuality and physical activity. Due to the topicality of sexual issues in stoma patients (Lin et al. 2023), all items of the SSES scale from the original version (Bekkers et al. 1996) are retained and evaluated in this study. Three factors were extracted using factor analysis and it is F3 that contains items that relate to stressful situations: sexuality, physical activity. Patients with stoma report sexual problems due to physiological changes in the body (stoma is a barrier to sex, they report disturbances in body perception, fear of sex, decrease in perceived sexual attraction) (Kimura et al. 2017). As a result of psychological changes which include reported feelings of embarrassment, guilt, changes in communication with spouses, and changes in the sexual attitudes of the partner leading to the end of the relationship there are also reports of seeking alternative ways of dealing with sex and the need for knowledge and professional counselling about sexuality with a stoma (Kandemir and Oskay 2017). It seems very appropriate to investigate these issues more closely.

According to Wang et al. (2022), the total score of the 28‐item SSES (range 28–140) is divided into three levels: a value ≤ 65 points indicates a low level of SE, a range of 66–102 points indicates a medium level of SE, and a value ≥ 103 points indicates a high level of SE. Higher scores indicate higher levels of perceived SE (Wang et al. 2022). In this study the SE results of stoma patients were more satisfactory than in China. Moreover, in the study of Wang et al. (2022), more respondents had low SE levels compared to our study, but more respondents had high SE levels than in this study. Using linear regression analysis, Wang et al. (2022) found that the main factors influencing SE of stoma patients were age and education level (higher education vs. primary school). In our study, respondent age negatively correlated with the total SE score, indicating that the total SE score decreases with increasing respondent age. The younger the respondent was at the time of stoma insertion, the higher the SE, particularly for respondents with ileostomy. Respondent age negatively correlates with Burden SE score for all respondents with stoma.

In a study by Wu et al. (2007), the older patients had lower levels of SE. Wu et al. (2007) found a negative correlation between the stoma care SE subscale and social SE. With higher levels of education, SE increased (Su et al. 2016; Wu et al. 2007). In our study, this was not confirmed in respondents with colostomy or ileostomy. Wu et al. (2007) found significant differences between men and women with stoma; it can be noted that men scored much higher SE. Consistent with the results are the findings in the present study, where men with colostomies had higher SE than women with colostomies. The results in a study by Jin et al. (2020) indicated a negative association between gender and SSES. The results of this study also showed that respondents had increased SE with a longer period since stoma insertion. Respondents who received pension had lower SE compared to those who received salary. Respondents who had a positive perception of the stoma had a higher SE. The association between positive acceptance of the stoma and SE in stoma care was also found in a study by Jin et al. (2020). Cultural and healthcare system factors may help explain these findings. In the Czech context, nurse‐led education and follow‐up care are well established, yet discussing emotional or sexual concerns remains sensitive. These aspects may influence how patients perceive and report their confidence in managing a stoma, and thus shape the overall factor structure and interpretation of self‐efficacy scores.

### 6.3 Implications for Clinical Practice and Research

The SSES demonstrates high reliability and validity for patients with intestinal stomas, specifically those with colostomies or ileostomies, and serves as a foundation for further research. It offers the plausibility for comparative studies among patients with other stoma types (e.g., urostomies). The scale's results provide insight into which areas of stoma‐related SE patients require additional support. This support may focus on theoretical knowledge or practical skills related to stoma care. Such findings are essential for improving quality of life and patient satisfaction with care.

In the Czech context, the SSES has not previously been applied. Its use is highly suitable for clinical practice, where nurses can assess the patient's level of SE, for example, prior to discharge, and plan targeted intervention strategies in stoma care accordingly. Moreover, the instrument can guide the education of other healthcare professionals involved in stoma management, such as home care or palliative care nurses. Utilisation of the SSES promotes and enhances clinical effectiveness, quality of care and patient satisfaction.

### 6.4 Limitations

The study focused on patients with stomas, namely those with colostomies or ileostomies. Other types of stomas were not considered in the research. Due to the number of 290 respondents, the research findings cannot be generalised to all patients with stomas. The results of the research are based on a subjective assessment by the respondents and therefore it has to be considered that the level of SE may change over time. The data were analysed in the available time using cross‐sectional analysis; thus, they were not assessed over time. Patients had been living with a stoma in place for more than 1 year. The study did not include patients in the immediate postoperative period. Complications that may affect self‐efficacy were not included in this study. Based on these results, a more in‐depth analysis would be useful for future research, particularly in a wider range of respondents with intestinal stoma, at different points of stoma insertion, and taking into consideration potential complications that may affect self‐efficacy. Additionally, we acknowledge that using the same sample for EFA and CFA may inflate model fit; however, due to our clinical objective (to compare SE structure between colostomy and ileostomy groups), we applied multi‐group CFA on these strata rather than a random split, constrained models parsimoniously, reported full fit and modification indices, examined measurement invariance and recommend future confirmation with independent samples or cross‐validation.

## 7 Conclusion

The Czech version of the SSES had excellent psychometric properties in patients with intestinal stoma, specifically colostomy or ileostomy. The analysis and its results showed that it is a reliable tool for use among patients with intestinal stomas. The use of the tool in clinical practice gives a basis for improving the quality of care for patients with stomas.

The SSES is a useful tool for assessing self‐efficacy by the patient, who can track the changing trend of self‐efficacy over time based on the SSES sub‐scores. The scale can also be used by nurses who can use the scale to identify and address individual patient needs based on the identification of current needs in self‐efficacy.

## Author Contributions

All authors made substantial contributions. Responsibility for the conception and design of the study was by L.M., E.R., K.K. and D.B. L.M. and K.K. participated in data collection. E.R., L.M. and D.B. participated in data analysis. The article was written with contributions from all authors. The study was supervised by L.M., E.R. and D.B. All authors approved the final version for submission. The author team includes the statistician Eva Reiterová, PhD.

## Funding

The authors have nothing to report.

## Disclosure

Declaration of generative AI and AI‐assisted technologies in the writing process: The research team declares there was no artificial intelligence or AI‐assisted technology used during any point of this study.

## Ethics Statement

The approval of the Ethics Committee of the Faculty of Health Sciences of Palacký University in Olomouc (UPOL‐253378/FZV‐2022) was obtained for the implementation of the research. Consent was obtained for the implementation of the research in the participating hospitals in Moravia; informed consent was obtained from patients in the participating stoma clubs who agreed to be involved in the study. Participation in the study was voluntary, and patients did not incur any obligations or claims.

## Conflicts of Interest

The authors declare no conflicts of interest.

## Supporting information


**Data S1:** jocn70168‐sup‐0001‐STROBE_checklist.docx.

## Data Availability

The data that support the findings of this study are available from the corresponding author upon reasonable request.
